# Editorial: Obesogens in the XXI century: emerging health challenges volume II

**DOI:** 10.3389/fendo.2023.1292334

**Published:** 2023-10-05

**Authors:** Ana C. A. Sousa, Govindan Malarvannan, Tomohiko Isobe, Luís Rato

**Affiliations:** ^1^ Department of Biology, School of Science and Technology, University of Évora, Évora, Portugal; ^2^ Comprehensive Health Research Centre (CHRC), University of Évora, Évora, Portugal; ^3^ Department of Pharmaceutical Sciences, Toxicological Centre, University of Antwerp, Antwerp, Belgium; ^4^ Japan Environment and Children’s Study Programme Office, National Institute for Environmental Studies, Ibaraki, Japan; ^5^ CICS-UBI - Health Sciences Research Centre, University of Beira Interior, Covilhã, Portugal; ^6^ Health School of the Polytechnic Institute of Guarda, Guarda, Portugal

**Keywords:** endocrine-disrupting chemicals (EDC), obesogens, obesity, public health, metabolic diseases

Obesity is a multifactorial disease considered by the World Health Organization (WHO) as one of the most important public health challenges of the 21^st^ century (1). Given its complex etiology, obesity prevention and treatment are very complex. It is, therefore, essential to understand all the drivers and molecular mechanisms associated with this complex disease. This Research Topic builds upon a previous one (2) and aims to further contribute with cutting-edge data from the molecular basis to the clinical perspective, including mechanisms, associated diseases, public health data, and recent breakthroughs in obesity related research.

Given its complex etiology, the causes of obesity are varied, but as strengthen by Peinado et al. eating behavior plays an important role, particularly the decisions on the type and variety of food. The authors performed a systematic review to assess the association between obesity and taste alterations. Despite some methodological limitations of the studies included, the authors concluded that there is a possible association between obesity and taste alterations. Given such results, it is plausible to suggest that obesity may “define” our consumption decisions. The authors also identified the limitations in the field and stressed the need for future longitudinal investigations using standardized methods to better describe the taste alterations and their interactions with other factors (3).

Another important shortcoming in the obesity field is associated with limitations in the implementation of successful health interventions in real world settings and the evaluation of the efficacy of those interventions as pinpointed by Øverby et al. To overcome this limitation and improve nutrition in the first 1000 days of life, the authors combined four effective dietary interventions into a single adapted digital resource (Nutrition Now). Their paper (4) describes the study protocol of a hybrid type 1 non-randomized trial to evaluate the effectiveness and implementation of evidence-based early-life nutrition interventions in a Norwegian community setting. The target population will be pregnant women and parents of 0–2-year-olds to which messages focusing on healthy dietary behaviors will be delivered with the goal of improving health.

Several aspects of the association between weight gain and diseases, including for example musculoskeletal disorders, remain to be clarified. He et al. evaluated the association between obesity and Achilles tendinopathy (AT), also known as Achilles tendinitis. This disease is associated with intense pain and is responsible for dysfunction and disability, leading to a significant decrease in both social and economic advantages. Yet, its risks factors are still not fully understood as the available observational epidemiological studies are controversial. To overcome these limitations, the authors conducted a Mendelian randomization (MR) study to screen for potential causal associations among ten putative risk factors, including body-mass index (BMI) and AT. The results of the MR study revealed a causal association between BMI and the risk of AT, suggesting that weight management might be a promising approach to prevent AT and therefore to reduce the associated burden. This study stress again the need to understand the risk factors to improve health.

In fact, identifying and understanding risks factors for the development of any disease is a unique opportunity for health improvement. As highlighted in the previous Research Topic (2), there are several chemical products known to be obesogens and therefore associated with weight gain. Bisphenol-A (BPA) is one of those suggested compounds. Heras-Gonzalez et al. examined the influence of BPA exposure measured in saliva together and daily physical activity on the obesity risk in schoolchildren from southern Spain. Their results demonstrated that children with higher levels of BPA in saliva had higher risk of overweight/obesity (OR=1.38, P95CI: 0.938-1.763), they also identified other factors that affected the risk of obesity, including body fat composition (OR = 10.77, P95CI: 5.89-19.70), not walking to and from school (OR = 1.38, P95CI: 1.005-1.902), lesser energy expenditure in sedentary activities (OR = 12.71, P95CI: 8.487-19.041), greater energy expenditure in sports (OR =1.62, P95CI: 1.171-2.223).

Besides these synthetic chemicals used directly in consumer products such as BPA, there are other molecules that might interact with the metabolic pathways, including for example Advanced Glycation End products (AGEs), or glycotoxins, formed by the non-enzymatic glycation of proteins, amino acids, and nucleic acids that occur e.g. during grilling, roasting, and broiling or frying of foods. These molecules are implicated in the development of diabetes complications and affect cardio-metabolic health. However, their role in obesity is still under debate as there are inconsistencies between the results of studies on the association of AGEs and obesity measurements. Jalil et al. performed a systematic review and meta-analysis in order to quantitatively analyze the results of studies that evaluated the association between circulating and dietary AGEs with obesity measurements among the adult population. Overall, the meta-analysis revealed an inverse association between circulating AGEs and body mass index among adults, yet the number of studies was limited. Further studies are necessary to completely understand the role of AGEs in obesity.

We hope that this Research Topic by compiling studies from the the possible risk factors to the consequences of obesity, contributes to improving our knowledge of this public health priority and may open new perspectives on this complex topic.

## Author contributions

AS: Writing – original draft, Writing – review & editing. GM: Writing – review & editing. TI: Writing – review & editing. LR: Writing – original draft, Writing – review & editing.

## References

[1] WHO. WHO factsheets: obesity and overweight. In: World health organization. (2016).

[2] Sousa ACA Malarvannan G Isobe T Rato L. Editorial: Obesogens in the XXI century: Emerging health challenges. Front Endocrinol 13 (2022). doi: 10.3389/fendo.2022.999908 PMC9393723 36004345

[3] Peinado BRR Frazão DR Bittencourt LO Souza-Rodrigues RDd Vidigal MTC da Silva DT. Is obesity associated with taste alterations? a systematic review. Front Endocrinol (2023) 14. doi: 10.3389/fendo.2023.1167119 PMC10273260 37334283

[4] Øverby NC Hillesund ER Helland SH Helle C Wills AK Lamu AN. Evaluating the effectiveness and implementation of evidence-based early-life nutrition interventions in a community setting a hybrid type 1 non-randomized trial – the Nutrition Now project protocol. Front Endocrinol (2023) 13. doi: 10.3389/fendo.2022.1071489 PMC9871808 36704042

